# A closer look at the time course of bodily responses to awe experiences

**DOI:** 10.1038/s41598-023-49681-2

**Published:** 2023-12-15

**Authors:** Ryota Takano, Michio Nomura

**Affiliations:** 1 Department of Social Psychology, The University of Tokyo, 7-3-1 Hongo, Bunkyo-ku, Tokyo, 113-0033 Japan; 2 Japan Society for the Promotion of Science, Tokyo, Japan; 3 Graduate School of Education, Kyoto University, Yoshida-Honmachi, Sakyo-ku, Kyoto, 606-8501 Japan

**Keywords:** Psychology, Emotion, Social neuroscience

## Abstract

The structure and function of awe have been examined by focusing on the *average* level of outcomes during awe experiences. In the present study, we tested the psychophysiological process of *experiencing* awe, focusing on time-series changes in skin conductance responses (SCRs), a moment-by-moment measure of sympathetic nervous responses, and pupil diameter, which is dilated or constricted through the sympathetic and parasympathetic nervous system. The responses were measured through an experiment where 77 Japanese university students watched emotional (awe, amusement, or neutral) videos while moving a joystick when they felt supernatural agency or non-agency, examining the underlying psychological processes. We found that experiencing awe was associated with frequent and steep changes in SCRs and frequent changes in pupil diameter. The joystick inclination, the perceptions of the supernatural, was kept at a high level from the start to the end of awe experiences. These results may reflect the psychophysiological processes of awe: the “fluctuation” of the sympathetic nervous system might underlie awe-specific experiences. Our findings shed new light on the mechanisms of the body-mind interaction in awe experiences.

## Introduction

Awe has been studied in religion, sociology, and philosophy, and more recently, its structure and function have received increasing attention in psychology^1–3^. Theoretically, awe is defined as an emotional response to perceptually and conceptually vast stimuli that transcend current frames of understanding^2^. Previous empirical research has revealed the average levels of physiological and psychological responses during experiences of awe, shedding light on whether these responses tend to be high or low^4–8^. However, little is known about the psychophysiological basis of *experiencing* awe, although several studies suggest that awe experiences are associated with changes in bodily responses^9,10^. For example, an awe-inspiring video exposure is linked to average changes in autonomic nervous system activity^8,11^. To understand the subtlety of these processes, in this study, we investigated time-series changes in bodily reactions during awe experiences with a particular focus on two physiological measures involving psychological processes: skin conductance responses (SCRs), which are a moment-by-moment measure of sympathetic nervous responses; and pupil diameter, which is dilated or constricted through the sympathetic and parasympathetic nervous system.

Awe experiences involve psychological processes including the quiet mind and simultaneously, an intense feeling that challenges one’s worldview^2,12^. In these views, experiences of awe are linked to both lower and higher levels of systematic nervous system responses^8,13^. On the one hand, previous studies found that awe experiences were associated with decreasing sympathetic nervous activation and increasing parasympathetic responses^8,11^. For example, the observation of slides displaying awe-inducing images (i.e., panoramic views) resulted in a reduction in the number of SCRs and an increase in respiratory sinus arrhythmia, indicative of vagal parasympathetic modulation of the heart rate, compared to images evoking amusement and neutral emotional states, respectively^8^. In addition, awe-inducing virtual reality (VR) videos led to an increase in the high frequency component of heart rate variability, which reflects cardiac parasympathetic modulation, more so than neutral videos^11^. On the other hand, awe was found to be related to increasing sympathetic nervous responses^11,13,14^. For instance, approximately half of participants experienced goose bumps as a sympathetic nervous response while watching awe-inspiring videos through VR, and self-reported awe ratings were positively correlated with the occurrence of goose bumps^14^. At a glance, these findings are controversial; however, it should be noted that these are results of physiological average changes. Thus far, no study of awe has considered a time-course perspective. In the realm of studies on other emotions, adopting a time-series perspective has proven efficacious in attaining a more nuanced understanding of the emotional phenomena. Levenson and his colleagues^15–17^ investigated emotional responses of participants while they watched a video by combining an affect rating dial and physiological measurements. Therefore, by focusing on time-series changes in physiological measures, which have high time resolution, we can deepen our understanding of *mental processes* of awe in terms of physiological patterns, beyond the discussion on whether a particular physiological response is high or low.

According to previous studies, awe experiences are specifically associated with changes in SCRs, which reflects phasic sympathetic responses toward stimuli^8,11^. For example, lower levels of number of SCRs were observed while looking at pictures in the awe condition than in other positive emotion conditions^8^. Meanwhile, individuals exhibited increased SCRs while experiencing awe through VR or video-based stimuli, which are thought to evoke intense feelings of awe^11,13,18^. Importantly, although SCRs have various indices, including time-course information (e.g., rise time, recovery time), that reflect the activation or deactivation of sympathetic nervous system responses, these measures have not been comprehensively examined with a focus on time-series contexts and the contents of awe stimuli.

Changes in pupil diameter are caused by the activation and deactivation of the sympathetic and parasympathetic nervous system^19^. Studies have demonstrated that changes in pupil diameter (i.e., both pupil dilation and constriction) are associated with attentional shifts and epistemic emotions, affective states that are related to knowledge and understanding toward stimuli such as surprise, indicating that pupil changes reflect internal processes toward external requirements in terms of initial information processing^20–22^. Importantly, from a theoretical perspective, awe is triggered by objects or persons that are beyond an individual’s existing knowledge structures, thereby involving the process of need for accommodation, which refers to the feeling that an individual is required to change existing mental structures to account for the gap between stimuli and one’s understanding^2,3,23^. Therefore, although no study has directly investigated the relationship between awe experiences and pupil diameter, prior studies suggest a possible link.

As a subjective phenomenon associated with the physiological responses during awe experiences, individuals might exhibit an increased tendency toward supernatural beliefs^3,24^. In general, people represent the supernatural in two ways: with agency and without agency^25^. Regarding the former, the supernatural includes God in Christianity and the spirits of gods that dwell in nature in Shintoism (e.g., mountains and rivers). Thus, this agency differs from the self. For the latter, the supernatural is not accompanied by agency, which is sometimes perceived as a continuation of the self^26,27^. For example, in Japanese nature worship, objects of reverence can be metaphysical, with nature itself being deified^26,28^. Based on these observations, we examined the differences in the relationship between the physiological responses during awe experiences and the two types of mental representations of the supernatural. Therefore, we assessed the time-series changes in the perceptions of the supernatural.

### Current research

This study aimed to explore the psychophysiological underpinnings of awe experiences by examining the temporal dynamics of bodily responses to awe-inspiring stimuli. We investigated time-series changes in SCRs and pupil diameter while participants viewed awe-inspiring videos. Furthermore, we examined awe-related subjective phenomena that occur in parallel with these physiological responses by measuring perceptions of the supernatural moment by moment. We exploratorily investigated whether specific patterns of changes in SCRs and pupil diameter during awe experiences are observed in relation to the perception of the supernatural from a time-series perspective.

## Methods

### Participants

Eighty right-handed Japanese university students participated in this study (Edinburgh Handedness Inventory score = 100)^29^. All participants reported that they had no history of psychiatric illness or neurological disorders. Three participants were excluded due to issues experienced in measuring their pupil diameter (more than 10% missing data). The final sample comprised 77 participants (39 men and 38 women; mean age = 21.44 years, standard deviation [*SD*] = 3.11). Our target sample size was determined using a priori power analysis (G*Power)^30^, which suggested that achieving 0.90 power at an α level of 0.05 for a medium-size within-subjects main effect of emotion manipulation required a sample of 36 participants. Given that we also investigated the relationships among variables, the required sample size was *N* = 72, as we doubled the suggested sample size (for details, please see supplemental materials S1). The study conformed to the principles expressed in the Declaration of Helsinki and was approved by the local ethics committee at the Graduate School of Education, Kyoto University (Ref-No. CPE-430). All participants provided written informed consent prior to their participation.

### Experimental design and procedure

**Figure 1:**
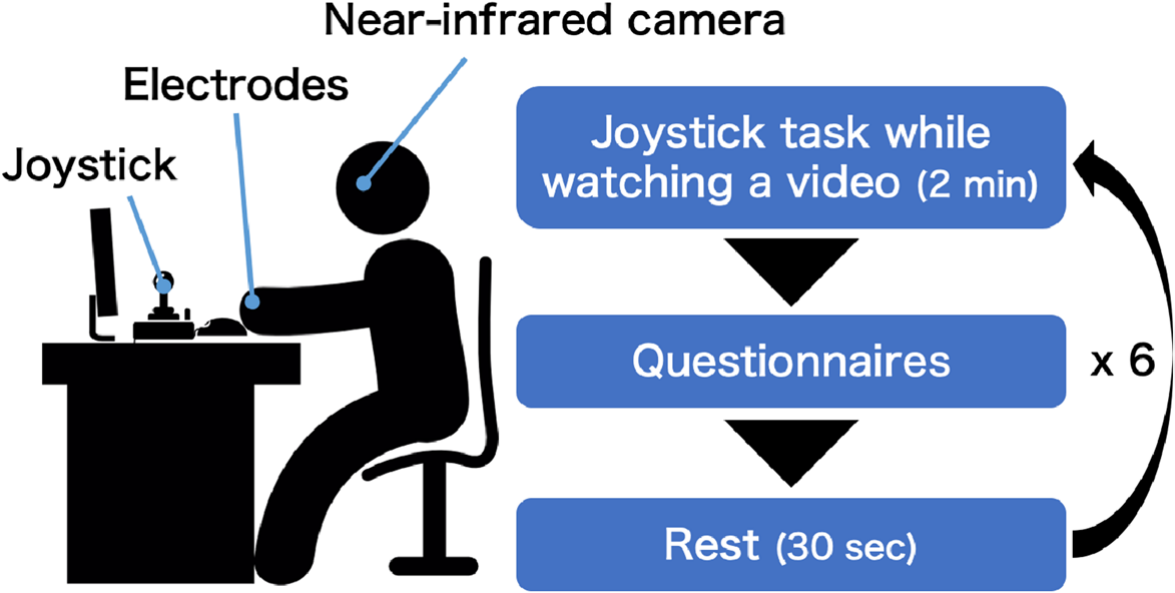
Experimental setting and procedure.

We employed a one-factor within-subjects design consisting of three levels of emotion manipulation (neutral, amusement, and awe). First, participants were outfitted with devices to measure physiological responses, followed by performing the main task after practice and a 30-s rest (Fig. 1). In the main task, participants completed a series of questionnaires after the joystick task while watching a 2-min video clip. We prepared two video clips for each emotion condition, and the order of the six video clips was randomized. All video clips were presented on a 21.5-inch full HD monitor using PsychoPy (PsychoPy3 [v2020.2.8])^31^. The experimenter recorded the temperature and humidity in the laboratory at the beginning of the main task. Participants were asked not to move their left arm or head to avoid signal artifacts.

#### Joystick task while watching a video

Participants watched a 2-min video, which has been used in previous studies^5,32^. Awe-inspiring clips comprised nature clips showing scenic views of nature and starry skies shot with time-lapse cameras. Amusement clips consisted of nature clips depicting animals performing humorous actions in nature. Neutral clips comprised nature clips showing a walk in the park. These video clips were presented at 1760 × 990 resolution. A separate pilot survey was conducted to validate the expected results (see supplemental materials S1).

To assess participants’ perceptions of the supernatural, they were asked to move a joystick (Logitech 3D Pro) in front of them to the right when they felt an “invisible presence” while watching the video. The experimenter instructed them to move the joystick deeper when they felt the presence more distinctly and told them that this invisible presence indicated (1) something larger than them, vast, and supernatural; and (2) something other than the sensory perceptions they would feel if they had gone to the place in the video (e.g., tactile sensations by the wind or smells from nature). These instructions were designed to prompt responses related to supernatural entities while minimizing participants’ resistance to questions of a religious or spiritual nature. The participants were instructed to answer intuitively to reduce demand characteristics. The joystick value was recorded from 0 for the upright to 0.5 for the most rightward position.

### Measures

#### Physiological measures

Electrodermal activity was recorded at 250 Hz from the index and middle fingertips of the left hand using 6 mm BIOPAC Ag–AgCl electrodes (TSD 203) and an amplifier (GSR 100C) with a 1.0 Hz high-pass filter. The electrodes were filled with 0.5% NaCl paste (GEL 101) and attached using Velcro tape. The number of non-specific SCRs, the amplitude of the signal, the rise time (duration until the SC response’s peak), and the recovery time (duration until the SC response returns to half its peak value) were extracted using the Python module neurokit2^33^. To exclude the SCRs induced by the start of the video presentation, SCRs that occurred for less than 10 s following the beginning of the video were not included in the analyses^34^. The values of the amplitude, rise time, and recovery time of SCRs were logarithmically transformed to fit a normal distribution after removing the excessive responses (above or below ± 3 *SD*). Pupil diameter was measured using a near-infrared video camera attached to the right side of the glasses (Gazo Co. Ltd.)^35^. The image series for pupil diameter was recorded with 640 × 480 resolution and 120-fps framerate. The pupil diameter data were preprocessed using the gazeR package (de-blinking, interpolation, and smoothing)^36^. We did not measure other physiological indices.

#### Questionnaires

After watching the video, participants were asked to rate their perceptions of the supernatural (agency and non-agency), emotional states, and senses of a small self and self-boundary using 7-point scales. The perceptions of the supernatural were measured using two items: “The ‘invisible presence’ in the previous video was the agency that transcended human beings (e.g., God or Buddha)” and “The ‘invisible presence’ in the previous video was not an entity, but something like the air that surrounded nature.” Emotional states were measured by asking how they had experienced 10 particular emotions, such as awe (mean scores of *ikei*, *ifu* [awe in Japanese], and wonder; α = 0.83) and amusement^9^. Participants were asked to select options that best represented their perceived self-size and perceived self-boundary through a choice of seven circles (Fig. S1A,B)^9^, to assess whether they felt a smaller sense of self and blurred self-boundary. We also administered other questionnaires for exploratory purposes (see supplemental materials S1).

### Statistical analyses

Data analyses were conducted using R (v. 4.3.1), an R package rstan (v. 2.21.2), and Python (v.3.6). In the Bayesian statistical analyses, we used the Markov Chain Monte Carlo (MCMC) method for parameter estimation and the $\widehat{R}$ statistic to diagnose convergence^37^. A coefficient was deemed statistically significant when the 95% posterior credible intervals (CIs) did not include zero. There was no meaningful change in the Bayesian statistical analyses results when we used the frequentist approach (Tables S1–S5).

#### Mean comparisons among conditions and relationships between variables

First, we compared the means of the SCR-related data (total number, amplitude, rise time, and recovery time), pupil diameter, and joystick data spanning 120 s (110 s for the SCR-related data) and the ratings of questionnaires between emotion conditions using hierarchical Bayesian models. In each model, we used temperature, humidity, and contrast-coding for the two emotion conditions (awe-contrast: awe = 2/3, amusement = − 1/3, neutral = − 1/3; amusement-contrast: awe = − 1/3, amusement = 2/3, neutral = − 1/3) as fixed effects with one random intercept for participants^9^. Regarding the physiological measurements, we examined whether there were effects of other emotions, habituation, or demographic heterogeneity because some previous studies have suggested that these factors may moderate the effects of awe^38–41^. We considered three models in addition to the simple models: control models, which controlled for the main effects of emotions other than awe that were elicited from the video (i.e., amusement and fear as general positive and negative emotions, respectively); order models, which included the interaction effects between the contrasts and the order in which the videos were presented within each emotion condition (first = − 1/2, second = 1/2); and demographic models with the interaction effects between awe-contrast and age and gender (the values of age were centered). Notably, these three models showed similar results as in the simple models concerning the effects of awe on each dependent variable. However, it is worth noting an exception, which pertains to the amplitude of SCRs when controlling for other emotions (Table S7). The results of these models are available in the supplemental materials S1. In this article, we report the results of the simple models.

Second, we conducted Bayesian regression analyses in each condition using generalized linear models. When the SCR peaks and recoveries were not observed for a participant in a condition, the SCR-related data for that cell were regarded as missing; thus, the missing data were handled using multiple imputation in the Multivariate Imputation by Chained Equations package with 100 imputed data sets^42^. There was no meaningful change in the results with the multiple imputation method when we only used the collected data without imputed data (see Supplemental Materials S1).

For these two analyses, using the brms package^43^, we obtained MCMC samples from four independent chains with 20,000 iterations, discarding the initial 10,000 samples for each chain as warm-ups. The posterior samples of 40,000 post-warmup draws indicated that the models converged ($\widehat{R}$ < 1.10; bulk and tail effective sample sizes > 1000).

#### Time-series analyses

Third, time-series changes in the number of SCRs, pupil diameter, and joystick data were examined using the state-space modeling, smoothed trend models with second-order differencing. We extracted the time-series data consisting of 120 time points (110 for the SCRs), one per second. The time-series data of the SCRs were the mean number of SCR peaks across participants per second. The time-series data of the pupil diameter and joystick data were obtained by calculating moving averages of the values per second. Details of the model equation are provided in the supplemental materials S1. In this model, we assumed a stepwise change in the degree of fluctuation between time points. This assumption allowed us to extract the standard deviation ${\upsigma }_{\upzeta }$, the degree of variation of the trend components (changes per unit time), which can capture the frequency of time-series changes as a parameter. We averaged this parameter between two video stimuli for each emotion condition and calculated the difference between awe/amusement and the other two conditions (i.e., ${\upsigma }_{{\text{dif}}({\text{awe}}-{\text{others}})}$ and ${\upsigma }_{{\text{dif}}({\text{amusement}}-{\text{others}})}$). We obtained MCMC samples from four independent chains of 20,000 iterations and discarded the initial 12,000 samples for each chain as warm-ups. The posterior samples of 32,000 post-warmup draws indicated that the models converged ($\widehat{{\varvec{R}}}$ < 1.10; bulk and tail effective sample sizes > 1000).

## Results

### Mean comparisons of physiological and self-report measurements

Mean comparisons using the hierarchical Bayesian method showed that the self-report measurements of awe (amusement) were significantly higher in the awe (amusement) condition than in the other conditions (awe: *M*_awe_ = 4.69, *M*_amusement_ = 2.16, *M*_neutral_ = 1.52, awe-contrast: *β* = 3.18, 95% CI [2.98, 3.37]; amusement: *M*_awe_ = 3.47, *M*_amusement_ = 4.72, *M*_neutral_ = 3.19, amusement-contrast: *β* = 1.53, 95% CI [1.18, 1.89]), indicating that the manipulation of emotion induction was successful (for other emotions, see Tables S1, S2, and S6). We found that the ratings of the small self and the self-boundary were significantly lower in the awe condition than in the other conditions (small self: *M*_awe_ = 2.17, *M*_amusement_ = 3.57, *M*_neutral_ = 4.10, awe-contrast: *β* = − 1.93, 95% CI [− 2.17, − 1.69]; self-boundary: *M*_awe_ = 2.80, *M*_amusement_ = 3.43, *M*_neutral_ = 4.41, awe-contrast: *β* = − 1.61, 95% CI [− 1.92, − 1.29]). The ratings of supernatural agency and non-agency were significantly higher in the awe condition than in the other conditions (agency: *M*_awe_ = 2.70, *M*_amusement_ = 1.56, *M*_neutral_ = 1.32, awe-contrast: *β* = 1.39, 95% CI [1.13, 1.64]; non-agency: *M*_awe_ = 5.25, *M*_amusement_ = 2.14, *M*_neutral_ = 2.41, awe-contrast: *β* = 2.86, 95% CI [2.50, 3.21]).

Regarding the mean comparisons of physiological measurements, as shown in Table 1, the total number and the amplitude of SCRs were significantly higher in the awe condition than in the other conditions (total number: *M*_awe_ = 2.38, *M*_amusement_ = 1.68, *M*_neutral_ = 0.91, awe-contrast: *β* = 1.47, 95% CI [1.08, 1.86]; amplitude: *M*_awe_ = − 0.61, *M*_amusement_ = − 0.70, *M*_neutral_ = − 0.79, awe-contrast: *β* = 0.18, 95% CI [0.04, 0.32]). The rise and recovery times of SCRs were the shortest in the awe condition (rise time: *M*_awe_ = 0.50, *M*_amusement_ = 0.56, *M*_neutral_ = 0.57, awe-contrast: *β* = − 0.07, 95% CI [− 0.13, − 0.02]; recovery time: *M*_awe_ = 0.64, *M*_amusement_ = 0.92, *M*_neutral_ = 1.10, awe-contrast: *β* = − 0.48, 95% CI [− 0.71, − 0.25]). The mean pupil diameter data in the amusement condition were the highest, followed by the awe and neutral conditions (*M*_awe_ = 3.54, *M*_amusement_ = 3.93, *M*_neutral_ = 3.41, awe-contrast: *β* = 0.13, 95% CI [0.09, 0.17], amusement-contrast: *β* = 0.53, 95% CI [0.48, 0.57]). Furthermore, the means of the joystick data (i.e., the perception of invisible presence) were significantly higher in the awe condition than in the other conditions (*M*_awe_ = 0.19, *M*_amusement_ = 0.04, *M*_neutral_ = 0.03, awe-contrast: *β* = 0.16, 95% CI [0.14, 0.18]). The effects of awe on the amplitude of SCRs were not significant when controlling for other emotions (awe-contrast: *β* = 0.14, 95% CI [− 0.02, 0.30]; Table S7).

**Table 1:** Results of mean comparisons of the skin conductance responses, pupil diameter, and joystick movement data between conditions using hierarchical Bayesian models.

Dependent variable	Parameter estimate	95% CI		R-hat	Bulk ESS	Tail ESS
		Lower	Upper			
Total number of SCRs						
Awe contrast	1.47	1.08	1.86	1.00	63,933	34,077
Amusement contrast	0.77	0.38	1.15	1.00	60,848	32,759
Amplitude of SCRs						
Awe contrast	0.18	0.04	0.32	1.00	22,972	28,473
Amusement contrast	0.10	− 0.04	0.25	1.00	22,177	27,000
Rise time of SCRs						
Awe contrast	− 0.07	− 0.13	− 0.02	1.00	49,218	33,077
Amusement contrast	− 0.02	− 0.08	0.03	1.00	48,436	33,053
Recovery time of SCRs						
Awe contrast	− 0.48	− 0.71	− 0.25	1.00	49,583	31,334
Amusement contrast	− 0.15	− 0.39	0.09	1.00	48,882	30,725
Pupil diameter						
Awe contrast	0.13	0.09	0.17	1.00	25,313	26,656
Amusement contrast	0.53	0.48	0.57	1.00	25,163	27,378
Joystick movement						
Awe contrast	0.16	0.14	0.18	1.00	63,183	32,842
Amusement contrast	0.01	− 0.01	0.03	1.00	63,271	33,736

Parameter estimate indicates expected a posteriori. Awe contrast (amusement contrast) was coded 2/3 for the awe (amusement) condition and − 1/3 for the other conditions. The values of amplitude, rise time, and recovery time of SCRs were logarithmically transformed. Random number seed was 1.

*SCRs* skin conductance responses, *CI* credible interval, *ESS* effective sample size.

### Relationships between physiological and self-report measurements

Bayesian regression analyses using generalized linear models with the multiple imputation method showed that, in the awe condition, the rise and recovery times of SCRs were positively associated with each other (*β* = 0.10, 95% CI [0.04, 0.16]). The number of SCRs was negatively related to the rise and recovery times of SCRs (rise time: *β* = − 4.12, 95% CI [− 6.71, − 1.49]; recovery time: *β* = − 0.87, 95% CI [− 1.39, − 0.34]), and the amplitude of SCRs was not associated with other SCR-related measures. In addition, positive relationships were noted between the amplitude of SCRs and the ratings of supernatural agency (*β* = 0.17, 95% CI [0.05, 0.29]). The means of joystick data (i.e., perceptions of the supernatural) were significantly positively related to the self-report measurements of awe (*β* = 0.04, 95% CI [0.02, 0.07]) and supernatural agency and non-agency (agency: *β* = 0.03, 95% CI [0.01, 0.05]; non-agency: *β* = 0.04, 95% CI [0.02, 0.06]). The relationship between supernatural agency ratings and non-agency ratings was not significant. Meanwhile, no significant relationships were found between pupil diameter and other variables.

### Time-series changes in SCRs, pupil diameter, and joystick reponses

**Figure 2:**
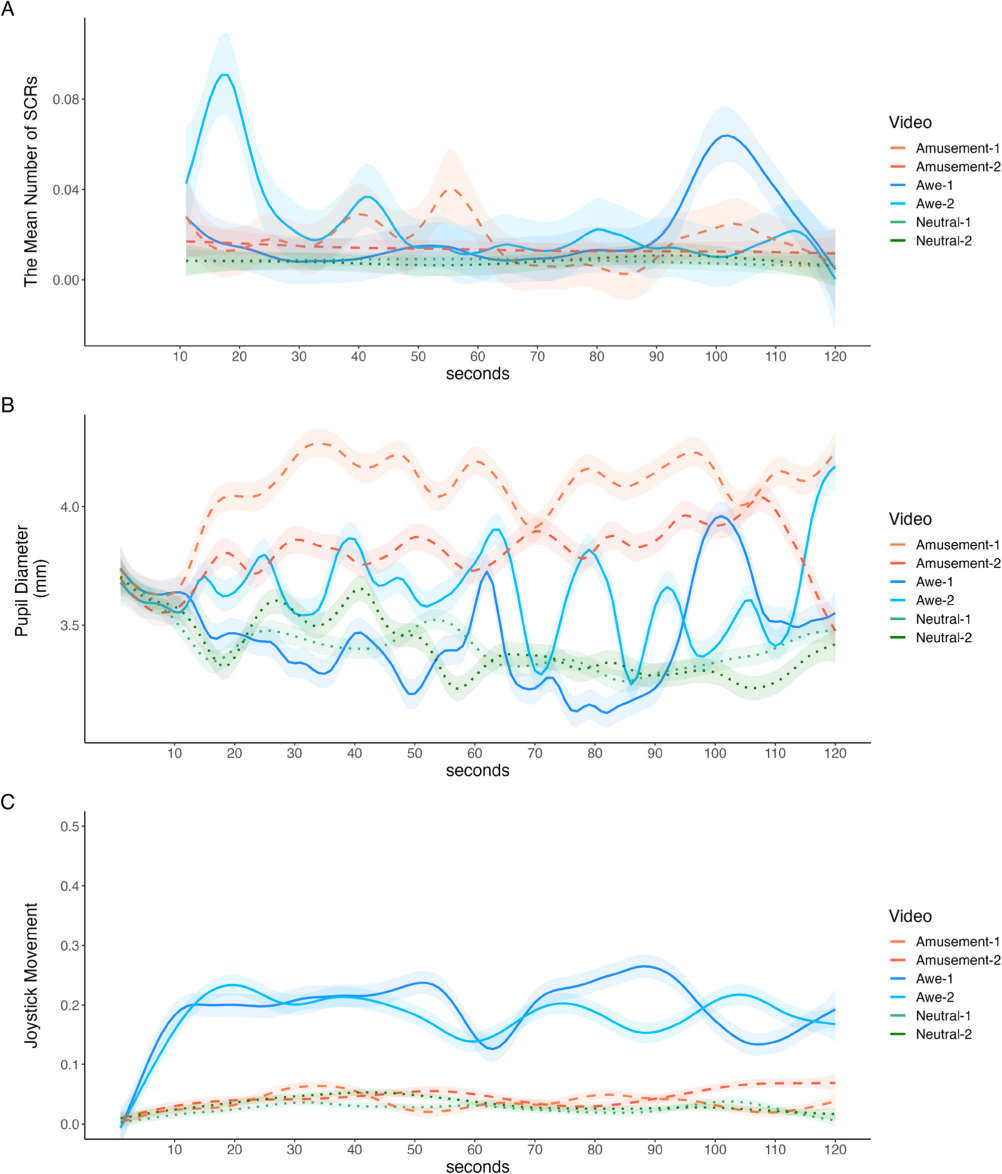
The plots for the time-series changes of (**A**) mean number of skin conductance responses, (**B**) pupil diameters, and (**C**) joystick movements using state-space modeling. The time-series line shows the expected a posteriori estimates of ${\upmu }_{{\text{t}}}$. The shaded areas indicate the 95% credible interval. *SCRs* skin conductance responses.

Figure 2 shows the time-series changes in the mean number of SCRs, pupil diameter, and joystick data while watching the video, using smoothed trend models with second-order differencing. We observed significant increases in the mean number of SCRs at approximately 90–110 s in the awe-1 video and 10–30 s in the awe-2 video (Fig. 2A, raw plots for the total number of SCRs per second are presented in Fig. S2). The views at both time points change to open (awe-1: from the ground to a waterfall; awe-2: from a place surrounded by trees to the top of trees; see awe-1.gif and awe-2.gif at https://osf.io/tcw3h/). The ${\upsigma }_{\upzeta }$ between conditions was larger in the awe condition than in the other conditions (${\upsigma }_{{\text{dif}}({\text{awe}}-{\text{others}})}$ = 0.003, 95% CI [0.001, 0.006]; ${\upsigma }_{{\text{dif}}({\text{amusement}}-{\text{others}})}$ = − 0.000, 95% CI [− 0.002, 0.002]), indicating remarkable changes in the number of SCRs during awe experiences. The time series of pupil diameter showed the most remarkable changes in dilation-constriction in the awe condition (Fig. 2B), consistent with the result that the ${\sigma }_{\zeta }$ in the awe condition was the largest (${\upsigma }_{{\text{dif}}({\text{awe}}-{\text{others}})}$ = 0.05, 95% CI [0.04, 0.07]; ${\upsigma }_{{\text{dif}}({\text{amusement}}-{\text{others}})}$ = − 0.02, 95% CI [− 0.03, 0.00]). This finding indicates that the changes in pupil dilation-constriction were most significant while watching awe-inspiring videos. Furthermore, it seemed that the inclination of the joystick gradually increased until approximately 10 s after the start of the awe-inspiring videos and then remained constant (Fig. 2C). However, it remained flat at roughly zero without any significant change in the amusement and neutral conditions.

## Discussion

In this study, we investigated the psychophysiological processes of *experiencing* awe, focusing on the time course of bodily responses during awe experiences. We examined time-series changes in SCR, pupil diameter, and joystick inclinations while participants watched awe-inspiring videos. The results showed that while experiencing awe, SCRs rose and recovered frequently and steeply, and the pupils frequently dilated and constricted. The inclination of the joystick, the perceptions of the supernatural, increased at the start of the awe-inspiring videos, which continued until the end.

In the awe condition, consistent with previous findings^8,11,14^, we observed both increased and decreased activations of SCRs and pupil responses, which are measures of the sympathetic nervous system. These results indicate that awe involves both affective arousal and relaxation, aligning with both intense and calming aspects of awe, in addition to reflecting dimensions of awe as “a complex emotion”. While prior research has suggested that awe is an ambiguous and complex psychological state^2,44,45^, the physiological evidence supporting this notion has been insufficient. Our findings are meaningful in suggesting that awe can be associated with both affective arousal and relaxation.

While experiencing awe, SCRs and pupil responses both activated and deactivated frequently and steeply, indicating that awe is associated with the “fluctuation” of systematic nervous system responses. Meanwhile, the perception of the supernatural increased from the start but did not change remarkably, suggesting that awe-related psychological phenomena arose continuously. Detecting the variability in physiological indicators during awe experiences is challenging in studies that solely depend on average values. Therefore, beyond the discussions based on the high or low averages of autonomic nervous system activity, in addition to being consistent with previous findings^8,13^, this is the first study to find the frequent fluctuation of sympathetic nervous activation and withdrawal simultaneously during awe experiences using the time-course lens.

Several recent studies have demonstrated that awe is related to self-transcendent and transformative experiences such as mindfulness, flow states, peak experiences, and mystical experiences, involving both high and low states of arousal^2,12,46,47^. Thus, it can be speculated that the fluctuation of the sympathetic nervous system could reflect the transformative process in awe experiences. Notably, the general implications of the fluctuation of the two physiological measurements (i.e., SCRs and pupil diameters) in psychophysiology are not fully understood owing to limited research in this area^20,35,48,49^. Further research is necessary to determine the specific psychological states associated with these physiological fluctuations.

We found an increased number or amplitude and decreased rise time of SCRs in the awe condition, compared to the neutral and amusement conditions. (Note that the effects of awe on the amplitude were not significant when controlling for other emotions.) Some studies showed that participants who looked at awe-inspiring pictures exhibited less numbers of SCRs^8^, while other studies as well as this study found that more immersive awe experiences through virtual reality or 2D videos were associated with more numbers of SCRs^11,14^. Given that these peak-related measures of SCRs are often observed in high arousal situations^50^, these findings indicate that more intense feelings of awe are accompanied by frequent and rapid physiological arousal.

Simultaneously, participants in the awe condition showed the shortest recovery time of SCRs. This pattern may reflect the process of quiet mind while experiencing awe, given that the fast recovery of the SCRs to baseline levels reflects the restoration of homeostatic balance through the reduction of arousal^48^; this may lead to the various positive effects of awe on psychological health, such as daily stress reduction^1^. We did not directly measure homeostatic balance under the stress conditions; however, the relationship between the short recovery time of SCRs and awe experiences is consistent with the transforming stress-related experiences by feeling awe.

Notably, we first showed that the SCRs during awe experiences were salient when participants watched the scenes where the views changed to be open. Previous research has shown that the perception of vastness is essential for the experience of awe; specifically, the spatially immense architectural feature is an essential structural factor in experiencing awe^51,52^. For example, Negami and Ellard^52^ investigated whether specific architectural features predict the emotion of awe, showing that spatial immensity and adornment uniquely predict the feeling of awe. Thus, certain structural characteristics of awe stimuli might cause physiological arousal changes, thereby arousing the experience of awe.

Participants’ pupils frequently dilated and constricted while experiencing awe. Researchers have argued that awe involves the need for accommodation, which is the process of requiring to update existing schemas^23^. Concurrently, awe is believed to involve the schema-liberation process; for example, an fMRI study showed that awe deactivates the left middle temporal gyrus that plays a role in applying current schema to events^32^. Meanwhile, frequent pupil dilation and constriction reflect an individual’s internal processes toward external requirements^20^. This internal shift lets individuals decouple from environmental requirements^53^. Based on these findings, one explanation for our results is that awe might involve the process of decoupling from transcendent stimuli as a means to liberate one’s schema.

Our findings have important implications, mainly for the growing science of positive emotions, and more generally, emotion theory or affective science^54–56^. Many recent studies on emotions have emphasized the treacherous characteristics of expressing specific emotions in natural language^57–60^. Prior studies have mainly operated under the assumption of emotional presence, which has led to comparatively limited scrutiny of the cognitive processes and mechanisms governing the manifestation of the emotion itself. This study attempted to reveal the mental processes while experiencing awe, based on a model-based time-series approach (i.e., state-space modeling), by using physiological measurements that have high temporal resolution. Thus, this study lends itself to theoretical and methodological contributions that could highlight important implications for future research on emotions.

### Limitations

Several limitations and directions for future research are worth noting. First, amusement is frequently employed as a comparative condition for studying awe. Nevertheless, this comparison primarily addresses the positive emotional facets of awe. Since the physiological measurements employed in this study might actively respond to the epistemic aspects of the awe experience, it is advisable to conduct additional research utilizing other epistemic emotions, such as surprise. This would contribute to a more nuanced and precise understanding of the distinctive characteristics associated with awe. Second, while we used emotional stimuli as previous studies have done to simplify the comparison of this study’s results with those of prior research, we did not completely control for the confounding effects of the visual characteristics (e.g., resolution, luminance, perspective changes). These characteristics may explain our findings since awe-videos may have higher resolution and luminance, and more drastic perspective changes compared to other videos. The creation of a validated database of emotion-eliciting video stimuli that control for these characteristics is necessary. Third, the findings are limited in terms of generalizability and heterogeneity. The positive and nature-based awe stimuli in this study were also the most commonly-used stimuli in previous studies, being independent of sample representativeness^5,32,61,62^. Consistent with this, exploratory investigations showed that even when controlling for other positive and negative emotions, the effects of awe on SCRs remained, except for the amplitude. Some studies suggest that psychophysiological processes of awe might be different depending on demographic characteristics, the elicitors of awe, and individual differences in emotion regulation and sensory perception^24,44,63,64^. Indeed, this study also showed that participants’ age moderated the effect of awe on decreasing recovery time of SCRs (the effect direction was the same, but the effect size was different; see supplemental materials S1 for details). It would be necessary to examine comprehensively whether and how the effects of awe are consistent across different stimuli and sample characteristics.

## Conclusions

In summary, this study is the first to demonstrate the time-series changes in bodily responses when experiencing awe. Through the lens of physiological changes captured moment by moment while experiencing awe, our findings provide a more nuanced understanding of the psychological mechanisms of awe, thereby offering essential insights into the mind–body interactions during emotional experiences.

### Supplementary Information

Supplementary Information.

## Data Availability

Data, analysis code, and materials are available at https://osf.io/tcw3h/.
