# Supplementary material for: A closer look at the time course of bodily responses to awe experiences
Source: Sci Rep. 2023 Dec 15;13:22506. doi: 10.1038/s41598-023-49681-2 (PMC10728215; doi:10.1038/s41598-023-49681-2)
Supplement: Supplementary file 1 — Supplementary Information. [file 41598_2023_49681_MOESM1_ESM.docx]

# Supplemental Materials for

# “A Closer Look at the Time-Course of Bodily Responses to Awe Experiences”

# Supplemental Methods

## Sample Size Determination

The number of participants was determined by conducting a priori power analysis using G*Power^1^ with α = 0.05, power = 0.90, number of groups = 1, number of measurements = 3, correlations among repeated measures = 0.5 (default estimator), nonsphericity correction ε = 1 (default estimator), and a medium effect size *f* = 0.25 (default estimator for a medium effect size) as we used a 3 (emotion conditions: neutral vs. amusement vs. awe) within-subject design. The analysis required a sample of 36 participants. In this study, we conducted a priori power analysis based on the analysis of variance (ANOVA) since we also conducted analyses using frequentist in addition to Bayesian statistics. The required sample size was *N* = 72 as we doubled the suggested sample size since we also investigated the relationships among variables using correlation and regression analyses.

## Self-Reported Validation of Stimuli

A pilot survey was conducted to validate the expected results. Thirty-two adults watched each video clip in a randomized order. They reported how intensely they felt 12 emotions, such as awe (mean scores of *ikei*, *ifu* [awe in Japanese], and wonder)^2^ and amusement, using 9-point scales (1 = *not at all*, 9 = *extremely*). The mean awe (amusement) scores were higher for videos of awe (amusement) condition than for videos of other conditions (awe: *M_awe_* = 6.38, 6.39; *M_amusement_* = 3.59, 4.19; *M_neutral_* = 2.84, 2.44; amusement: *M_awe_* = 4.31, 4.84; *M_amusement_* 5.88, 6.38; *M_neutral_* = 4.78, 4.91).

## Other Questionnaires

In addition to the perceptions of the supernatural agency and non-agency, emotional states, and senses of a small self and self-boundary, participants were also asked to rate their perceptions of uncertainty, subjective stress, sense of liberation, and state levels of life satisfaction using 7-point scales after watching a video. The perception of uncertainty was measured using the item, “how uncertain did you feel while watching the previous video?”^3^. The perception of subjective stress was measured using four items (α = .78)^4,5^. The sense of liberation was measured using the item, “how did you feel a sense of liberation from yourself and your problems?” The state level of life satisfaction was measured using five items (α = .88)^6,7^. Results regarding these questionnaires are available at Tables S1-S6.

## Models Including the Order of the Video or Control Variables

Regarding mean comparisons of physiological measurements among conditions, we also considered the models controlling for the self-report measurements of amusement and fear (“control models”), those with the effects of the present order of the videos (“order models”), and those with the effects of demographic variables (i.e., age and gender) as follows:

Control models:

$$y=\alpha_{subject}+{\beta_{0}+\beta}_{1}awe\_contrast+\beta_{2}amusement\_contrast+\beta_{3}amusement+\beta_{4}fear$$

Order models:

$$y=\alpha_{subject}+\beta_{0}+\beta_{1}awe\_contrast+\beta_{2}amusement\_contrast+\beta_{3}order+\beta_{4}awe\_contrast\times order+\beta_{5}amusement\_contrast\times order$$

Demographic models:

$$y=\alpha_{subject}+\beta_{0}+\beta_{1}awe\_contrast+\beta_{2}amusement\_contrast+\beta_{3}age+\beta_{4}gender+\beta_{5}awe\_contrast\times age+\beta_{6}awe\_contrast\times gender$$

Where the terms $\alpha_{subject}$ represent random intercept for each individual. Note that the distribution of each parameter and fixed effects of humidity and temperature are omitted. We compared these three models with the simple models which only include the effects of awe-contrast and amusement-contrast (and humidity and temperature) based on their wAIC (Widely Applicable Information Criteria)^8^. Because wAIC varies depending on the random number seed of the MCMC, we ran 20 sampling runs per model, shifting the random number seed from 1 to 20. We reported the results of parameter estimation for the model with the lowest wAIC value among the models with significantly lower wAIC than the simple models (Table S7).

## Smoothed Trend Modeling

We used smoothed trend models with second-order differencing to examine time-series changes in pupil diameter, and joystick data. The model equation was as follows:

$$\left( \mu_{t}-\mu_{t-1} \right)-\left( \mu_{t-1}-\mu_{t-2} \right)=\zeta_{t}, \zeta_{t}\sim\mathrm{Normal}\left( 0, \sigma_{\zeta}^{2} \right)$$

$$y_{t,i}=\mu_{t}+\epsilon_{t}, \epsilon_{t}\sim\mathrm{Normal}\left( 0, \sigma_{\epsilon}^{2} \right)$$

The upper indicates a state equation representing the time-series changes in the number of SCRs, pupil diameter, or joystick data at the group level. The lower displays an observation equation where individual data are generated by appending observation errors to the group-level SCRs, pupil diameter, or joystick data at each time point. In this model equation, $t$ is the second (i.e., each timepoint), $i$ is the participant’s identification (ID), and $y_{t,i}$ is the number of SCRs, pupil diameter, and joystick data for each participant at each time point. The parameter $\mu_{t}$ represents the group-level state at each time point. The $\zeta_{t}$ and $\epsilon_{t}$ represent the disturbance and observation error terms, respectively. A normal white noise and a normal distribution were assumed to be generated with mean $\mu_{\zeta}=0$ and variance $\sigma_{\zeta}^{2}$ for the disturbance and with mean $\mu_{\epsilon}=0$ and variance $\sigma_{\epsilon}^{2}$ for the observation error, respectively.

# Supplemenral Results

## Bayesian Regression Analyses Without the Multiple Imputation Method

Baysian regression analyses without the multiple imputation method showed similar results with those analyses using with the method. In the awe condition, positive relationships were noted between the rise and recovery time of SCRs (*β =* 0.11, 95% CI [0.05, 0.16]), amplitude of SCRs and the ratings of supernatural agency (*β =* 0.17, 95% CI [0.06, 0.29]), and the means of joystick data and the self-report measures of awe and supernatural agency and non-agency (awe: *β =* 0.04, 95% CI [0.02, 0.07]; agency: *β =* 0.03, 95% CI [0.01, 0.05]; non-agency: *β =* 0.04, 95% CI [0.02, 0.06]). The number of SCRs was negatively related to the rise and recovery time of SCRs (rise time: *β =* -4.69, 95% CI [-7.41, -1.93]; recovery time: *β =* -0.90, 95% CI [-1.47, -0.33]).

## Three Additional Models: Control, Order, and Demographic Modles

Regarding the total number, amplitude, and recovery time of SCRs, the control models provided better fits than the simple models (Table S7). The control models showed that the effects of awe-contrast remained significant when controlling for the self-report measurements of amusement and fear (total number: *β =* 1.24, 95% CI [0.81, 1.66]; recovery time: *β =* -0.38, 95% CI [-0.63, -0.12]), except for the amplitude of SCRs (*β =* 0.14, 95% CI [-0.02, 0.30]).

In addition, for the total number and rise time of SCRs and pupil diameter, the order models provided better fits than the simple models (Table S7). In these models, the effects of order were significant on these three measurements (total number: *β =* -0.51, 95% CI [-0.82, -0.20]; rise time: *β =* 0.05, 95% CI [0.01, 0.09]; pupil diameter: *β =* -0.13, 95% CI [-0.16, -0.10]), and the interaction effects between awe-contrast and order were not significant; however, those of awe-contrast remained significant, indicating that there might be habituation effects irrelevant to the emotion conditions.

We also found that demographic models were more fitted than the simple model for the recovery time of SCRs. There were significant interaction effects between awe-contrast and age on recovery time (*β =* -0.08, 95% CI [-0.13, -0.02]); specifically, the effects of awe-contrast on recovery time were more evident among older participants (older [+1 *SD*]: *β =* -0.74, 95% CI [-1.03, -0.46], younger [-1 *SD*]: *β =* -0.26, 95% CI [-0.54, 0.02]).

## Figure S1.

*Perceived (A) Small-Self*^9^ *and (B) Self-Boundary Scales*^2^*.*

*Note*. Participants were asked to select options that best represented their perceived self-size or self-boundary. Specific instructions for perceived self-size were, “While watching the video, how small or large did you feel yourself to be? Please select the one that best describes the size of your own presence from the circles shown below.” Specific instructions for perceived self-boundary were, “While watching the video, how clear or vague did you feel yourself to be in the moment? Please select the one that best describes the clarity of your own presence in the place from the circles shown below.”

Figure S2.

*Plots for the Total Numbers of SCRs Across Participants per Second in Each Video (A: Awe-1, B: Awe-2, C: Amusement-1. D: Amusement-2, E: Neutral-1, F: Neutral-2).*

*
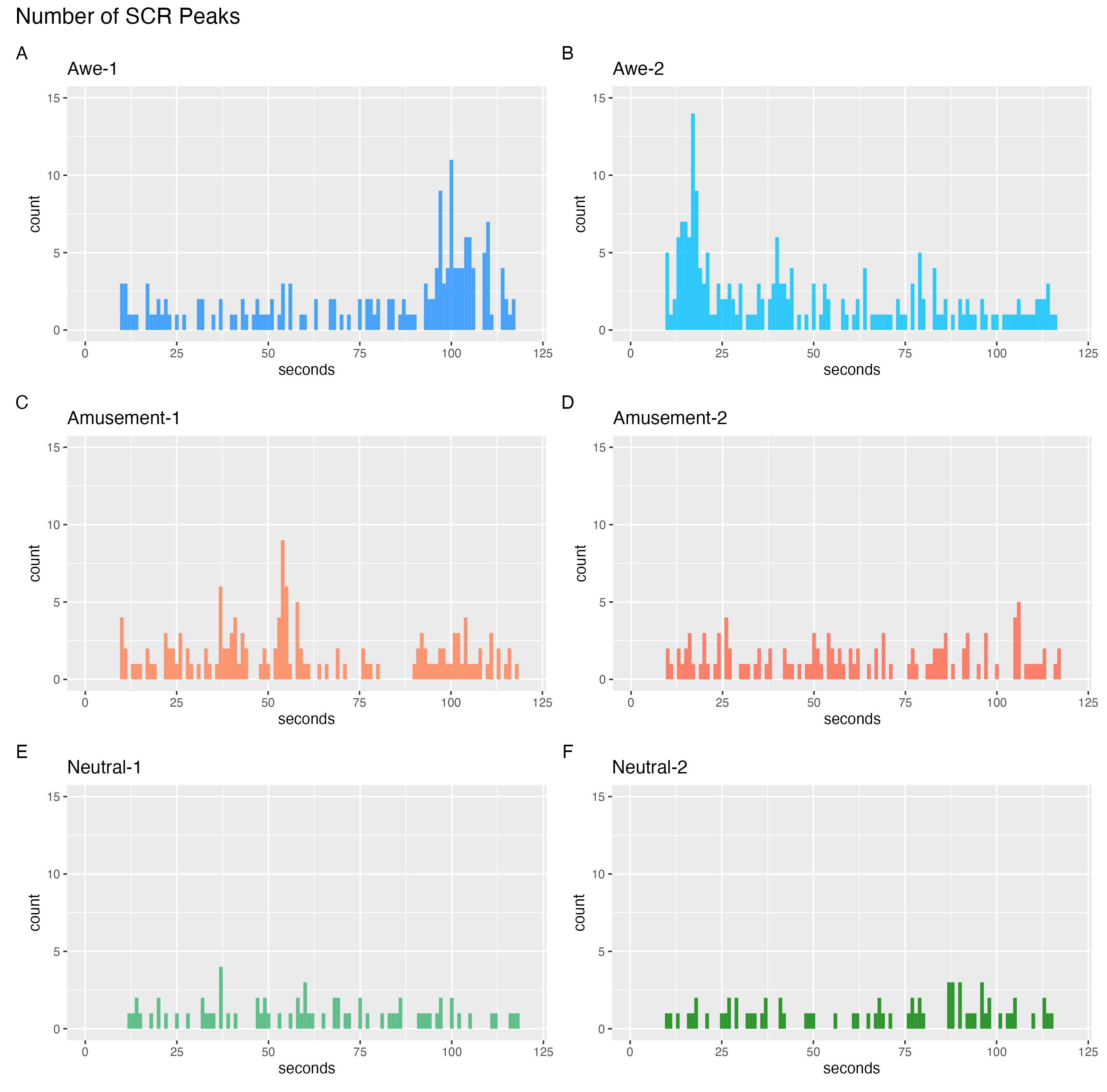
*

Table S1.

*Mean Comparisons of Each Variable Between Conditions Using Linear Mixed-Effects Models*

| Dependent Variables | | Estimate | 95% CI | | *p*-value |
| --- | --- | --- | --- | --- | --- |
|  |  |  | Lower | Upper |  |
| Total Number of SCRs | |  |  |  |  |
|  | Awe_contrast | 1.47 | 1.09 | 1.86 | <.001 |
|  | Amusement_contrast | 0.77 | 0.38 | 1.15 | <.001 |
| Amplitude of SCRs | |  |  |  |  |
|  | Awe_contrast | 0.18 | 0.05 | 0.32 | .009 |
|  | Amusement_contrast | 0.10 | –0.04 | 0.25 | .162 |
| Rise Time of SCRs | |  |  |  |  |
|  | Awe_contrast | –0.07 | –0.13 | –0.02 | .004 |
|  | Amusement_contrast | –0.02 | –0.08 | 0.03 | .440 |
| Recovery Time of SCRs | |  |  |  |  |
|  | Awe_contrast | –0.48 | –0.71 | –0.25 | <.001 |
|  | Amusement_contrast | –0.15 | –0.39 | 0.09 | .231 |
| Pupil Diameter | |  |  |  |  |
|  | Awe_contrast | 0.13 | 0.09 | 0.17 | <.001 |
|  | Amusement_contrast | 0.53 | 0.48 | 0.57 | <.001 |
| Joystick Movement | |  |  |  |  |
|  | Awe_contrast | 0.16 | 0.14 | 0.18 | <.001 |
|  | Amusement_contrast | 0.01 | –0.01 | 0.03 | .208 |
| Self-report Awe | |  |  |  |  |
|  | Awe_contrast | 3.18 | 2.98 | 3.37 | <.001 |
|  | Amusement_contrast | 0.64 | 0.44 | 0.83 | <.001 |
| Self-report Amusement | |  |  |  |  |
|  | Awe_contrast | 0.27 | –0.08 | 0.62 | .127 |
|  | Amusement_contrast | 1.53 | 1.18 | 1.88 | <.001 |
| Small Self | |  |  |  |  |
|  | Awe_contrast | –1.93 | –2.17 | –1.69 | <.001 |
|  | Amusement_contrast | –0.53 | –0.77 | –0.29 | <.001 |
| Self-boundary | |  |  |  |  |
|  | Awe_contrast | –1.61 | –1.92 | –1.29 | <.001 |
|  | Amusement_contrast | –0.98 | –1.29 | –0.67 | <.001 |
| Perceived Uncertainty | |  |  |  |  |
|  | Awe_contrast | 0.53 | 0.20 | 0.85 | .001 |
|  | Amusement_contrast | 1.89 | 1.57 | 2.21 | <.001 |
| Life Satisfaction | |  |  |  |  |
|  | Awe_contrast | –0.03 | –0.11 | 0.05 | .472 |
|  | Amusement_contrast | –0.06 | –0.15 | 0.02 | .133 |
| Subjective Stress | |  |  |  |  |
|  | Awe_contrast | –0.12 | –0.53 | 0.29 | .571 |
|  | Amusement_contrast | 0.12 | –0.30 | 0.53 | .582 |
| Supernatural Agency | |  |  |  |  |
|  | Awe_contrast | 1.39 | 1.14 | 1.64 | <.001 |
|  | Amusement_contrast | 0.64 | –0.00 | 0.51 | .052 |
| Supernatural Non-gency | |  |  |  |  |
|  | Awe_contrast | 2.86 | 2.50 | 3.21 | <.001 |
|  | Amusement_contrast | –0.27 | –0.62 | 0.09 | .142 |
| Self-report *Ikei* | |  |  |  |  |
|  | Awe_contrast | 3.55 | 3.30 | 3.80 | <.001 |
|  | Amusement_contrast | –0.10 | –0.35 | 0.15 | .440 |
| Self-report *Ifu* | |  |  |  |  |
|  | Awe_contrast | 2.91 | 2.65 | 3.17 | <.001 |
|  | Amusement_contrast | 0.11 | –0.16 | 0.37 | .422 |
| Self-report Wonder | |  |  |  |  |
|  | Awe_contrast | 3.10 | 2.80 | 3.41 | <.001 |
|  | Amusement_contrast | 1.89 | 1.58 | 2.19 | <.001 |
| Self-report Happiness | |  |  |  |  |
|  | Awe_contrast | 0.04 | –0.29 | 0.37 | .817 |
|  | Amusement_contrast | –0.23 | –0.56 | 0.11 | .181 |
| Self-report Fear | |  |  |  |  |
|  | Awe_contrast | 1.51 | 1.20 | 1.81 | <.001 |
|  | Amusement_contrast | 1.11 | 0.81 | 1.42 | <.001 |
| Self-report Anxiety | |  |  |  |  |
|  | Awe_contrast | 1.00 | 0.67 | 1.32 | <.001 |
|  | Amusement_contrast | 0.64 | 0.32 | 0.97 | <.001 |
| Self-report Respect | |  |  |  |  |
|  | Awe_contrast | 2.50 | 2.23 | 2.76 | <.001 |
|  | Amusement_contrast | 0.11 | –0.15 | 0.38 | .393 |
| Self-report Sadness | |  |  |  |  |
|  | Awe_contrast | –0.04 | –0.26 | 0.18 | .702 |
|  | Amusement_contrast | –0.09 | –0.31 | 0.13 | .444 |
| Self-report Anger | |  |  |  |  |
|  | Awe_contrast | 0.13 | –0.04 | 0.30 | .134 |
|  | Amusement_contrast | 0.56 | 0.40 | 0.73 | <.001 |
| *Note*. Awe_contrast (Amusement_contrast) was coded 2/3 for the awe (amusement) condition and –1/3 for the other conditions; CI: confidence interval | | | | | |

Table S2.

*Pairwise Comparisons of Awe Versus Amusement and Neutral Conditions on Each* *Variable Using ANOVA*

| Variables | Condition | *Mean* | *SD* | Comparisons with Awe | | |
| --- | --- | --- | --- | --- | --- | --- |
|  |  |  |  | *t* | *p* | Cohen's *d* |
|  |  |  |  |  |  |  |
| Self-report Awe | Awe | 4.69 | 1.43 | － | － | － |
|  | Amusement | 2.16 | 1.04 | -20.73 | <.001 | -2.36 |
|  | Neutral | 1.52 | 0.79 | -28.00 | <.001 | -3.19 |
| Self-report Amusement | Awe | 3.47 | 1.84 | － | － | － |
|  | Amusement | 4.72 | 1.90 | 6.05 | <.001 | 0.69 |
|  | Neutral | 3.19 | 1.84 | -1.60 | .111 | -0.18 |
| Small Self | Awe | 2.17 | 1.04 | － | － | － |
|  | Amusement | 3.57 | 1.35 | 10.86 | <.001 | 1.24 |
|  | Neutral | 4.10 | 1.13 | 15.29 | <.001 | 1.74 |
| Self-boundary | Awe | 2.80 | 1.50 | － | － | － |
|  | Amusement | 3.43 | 1.57 | 3.91 | <.001 | 0.45 |
|  | Neutral | 4.41 | 1.49 | 8.83 | <.001 | 1.01 |
| Perceived Uncertainty | Awe | 2.89 | 1.57 | － | － | － |
|  | Amusement | 4.25 | 1.77 | 7.84 | <.001 | 0.89 |
|  | Neutral | 2.36 | 1.60 | -3.56 | <.001 | -0.41 |
| Life Satisfaction | Awe | 4.28 | 1.18 | － | － | － |
|  | Amusement | 4.24 | 1.18 | -0.63 | 1.000 | -0.07 |
|  | Neutral | 4.31 | 1.20 | 0.43 | 1.000 | 0.05 |
| Perceived Stress | Awe | 4.01 | 1.06 | － | － | － |
|  | Amusement | 4.06 | 1.09 | 0.81 | 1.000 | 0.09 |
|  | Neutral | 4.02 | 1.09 | 0.50 | 1.000 | 0.06 |
| Supernatural Agency | Awe | 2.70 | 1.85 | － | － | － |
|  | Amusement | 1.56 | 1.13 | -7.20 | <.001 | -0.82 |
|  | Neutral | 1.32 | 0.89 | -9.39 | <.001 | -1.07 |
| Supernatural  No-agency | Awe | 5.25 | 1.92 | － | － | － |
|  | Amusement | 2.14 | 1.66 | -17.17 | <.001 | -1.96 |
|  | Neutral | 2.41 | 1.96 | -14.86 | <.001 | -1.69 |
| Self-report *Ikei* | Awe | 5.29 | 1.54 | － | － | － |
|  | Amusement | 1.65 | 1.10 | -25.95 | <.001 | -2.96 |
|  | Neutral | 1.75 | 1.35 | -24.38 | <.001 | -2.78 |
| Self-report *Ifu* | Awe | 4.31 | 1.92 | － | － | － |
|  | Amusement | 1.51 | 1.00 | -17.33 | <.001 | -1.97 |
|  | Neutral | 1.40 | 0.83 | -18.78 | <.001 | -2.14 |
| Self-report Wonder | Awe | 4.53 | 1.84 | － | － | － |
|  | Amusement | 3.30 | 1.94 | -6.57 | <.001 | -0.75 |
|  | Neutral | 1.42 | 0.91 | -21.29 | <.001 | -2.43 |
| Self-report Happiness | Awe | 3.44 | 1.84 | － | － | － |
|  | Amusement | 3.17 | 1.94 | -1.46 | .441 | -0.17 |
|  | Neutral | 3.40 | 1.92 | -0.23 | .816 | -0.03 |
| Self-report Fear | Awe | 2.89 | 1.77 | － | － | － |
|  | Amusement | 2.50 | 1.94 | -2.07 | .040 | -0.24 |
|  | Neutral | 1.38 | 0.92 | -10.97 | <.001 | -1.25 |
| Self-report Anxiety | Awe | 2.96 | 1.74 | － | － | － |
|  | Amusement | 2.61 | 1.93 | -2.09 | .039 | -0.24 |
|  | Neutral | 1.96 | 1.50 | -5.91 | <.001 | -0.67 |
| Self-report Respect | Awe | 3.98 | 1.95 | － | － | － |
|  | Amusement | 1.59 | 1.07 | -15.14 | <.001 | -1.73 |
|  | Neutral | 1.48 | 1.11 | -15.24 | <.001 | -1.74 |
| Self-report Sadness | Awe | 1.59 | 1.07 | － | － | － |
|  | Amusement | 1.55 | 1.26 | -0.44 | 1.000 | -0.05 |
|  | Neutral | 1.63 | 1.18 | 0.38 | 1.000 | 0.04 |
| Self-report Anger | Awe | 1.19 | 0.62 | － | － | － |
|  | Amusement | 1.63 | 1.25 | 4.19 | <.001 | 0.48 |
|  | Neutral | 1.07 | 0.30 | -2.54 | .012 | -0.29 |
| *Note*. Corrections for multiple comparisons were done using the Holm method. | | | | | | |

Table S3.

*Zero-order Correlations among Variables (Awe Condition)*

| Variables | 2 | 3 | 4 | 5 | 6 | 7 | 8 | 9 | 10 | 11 | 12 | 13 | 14 | 15 |
| --- | --- | --- | --- | --- | --- | --- | --- | --- | --- | --- | --- | --- | --- | --- |
| 1. Number of SCRs | 0.15 | -0.40 | -0.37 | 0.12 | -0.31 | -0.03 | 0.04 | -0.12 | -0.12 | 0.03 | -0.20 | -0.02 | 0.08 | -0.24 |
|  | (.24) | (<.001) | (<.001) | (.30) | (.01) | (.78) | (.76) | (.29) | (.29) | (.77) | (.07) | (.85) | (.46) | (.04) |
| 2. Amplitude of SCRs |  | 0.14 | -0.22 | 0.08 | 0.16 | 0.22 | 0.07 | -0.04 | 0.09 | 0.15 | 0.17 | -0.12 | 0.36 | 0.19 |
|  |  | (.37) | (.09) | (.54) | (.19) | (.08) | (.58) | (.74) | (.46) | (.24) | (.18) | (.35) | (<.001) | (.14) |
| 3. Rise Time of SCRs |  |  | 0.43 | 0.01 | 0.23 | 0.19 | 0.12 | -0.13 | 0.19 | 0.08 | -0.04 | 0.13 | 0.01 | 0.22 |
|  |  |  | (<.001) | (.95) | (.06) | (.14) | (.34) | (.32) | (.14) | (.53) | (.74) | (.32) | (.91) | (.08) |
| 4. Recovery Time of SCRs |  |  |  | 0.03 | 0.09 | 0.06 | -0.01 | 0.07 | 0.06 | 0.02 | 0.00 | -0.14 | -0.04 | 0.14 |
|  |  |  |  | (.81) | (.48) | (.61) | (.95) | (.56) | (.63) | (.88) | (.99) | (.25) | (.76) | (.25) |
| 5. Pupil Diameter |  |  |  |  | -0.16 | -0.08 | -0.09 | 0.05 | -0.02 | -0.05 | -0.13 | 0.14 | -0.15 | -0.07 |
|  |  |  |  |  | (.15) | (.50) | (.42) | (.67) | (.89) | (.67) | (.26) | (.24) | (.19) | (.57) |
| 6. Joystick Movement |  |  |  |  |  | 0.37 | 0.01 | -0.23 | -0.05 | 0.13 | 0.12 | -0.08 | 0.31 | 0.47 |
|  |  |  |  |  |  | (<.001) | (.95) | (.05) | (.64) | (.27) | (.31) | (.49) | (.01) | (<.001) |
| 7. Awe-ratings |  |  |  |  |  |  | 0.31 | -0.16 | 0.06 | 0.35 | -0.04 | 0.04 | 0.37 | 0.43 |
|  |  |  |  |  |  |  | (.01) | (.16) | (.61) | (<.001) | (.70) | (.76) | (<.001) | (<.001) |
| 8. Amusement-ratings |  |  |  |  |  |  |  | 0.08 | 0.02 | -0.05 | 0.17 | -0.03 | -0.01 | 0.05 |
|  |  |  |  |  |  |  |  | (.50) | (.84) | (.64) | (.14) | (.81) | (.92) | (.65) |
| 9. Small Self |  |  |  |  |  |  |  |  | 0.50 | -0.20 | 0.16 | -0.16 | -0.30 | -0.06 |
|  |  |  |  |  |  |  |  |  | (<.001) | (.07) | (.15) | (.17) | (.01) | (.61) |
| 10. Self-boundary |  |  |  |  |  |  |  |  |  | -0.19 | 0.12 | -0.09 | -0.12 | -0.04 |
|  |  |  |  |  |  |  |  |  |  | (.10) | (.31) | (.44) | (.29) | (.74) |
| 11. Perceived Uncertainty |  |  |  |  |  |  |  |  |  |  | 0.05 | -0.01 | 0.49 | 0.1 |
|  |  |  |  |  |  |  |  |  |  |  | (.69) | (.94) | (<.001) | (.41) |
| 12. Life Satisfaction |  |  |  |  |  |  |  |  |  |  |  | -0.5 | -0.02 | -0.08 |
|  |  |  |  |  |  |  |  |  |  |  |  | (<.001) | (.86) | (.48) |
| 13. Subjective Stress |  |  |  |  |  |  |  |  |  |  |  |  | 0.03 | 0.05 |
|  |  |  |  |  |  |  |  |  |  |  |  |  | (.80) | (.68) |
| 14. Supernatural Agency |  |  |  |  |  |  |  |  |  |  |  |  |  | 0.10 |
|  |  |  |  |  |  |  |  |  |  |  |  |  |  | (.40) |
| 15. Supernatural Non-agency |  |  |  |  |  |  |  |  |  |  |  |  |  |  |
| *Note*. Values in parenthesis indicate *p*-value. | | |  |  |  |  |  |  |  |  |  |  |  |  |

Table S4.

*Zero-order Correlations among Variables (Amusement Condition)*

| Variables | 2 | 3 | 4 | 5 | 6 | 7 | 8 | 9 | 10 | 11 | 12 | 13 | 14 | 15 |
| --- | --- | --- | --- | --- | --- | --- | --- | --- | --- | --- | --- | --- | --- | --- |
| 1. Number of SCRs | 0.07 | -0.31 | -0.45 | 0.26 | -0.02 | -0.07 | -0.06 | -0.13 | -0.11 | -0.19 | -0.20 | -0.03 | -0.06 | 0.05 |
|  | (.59) | (.02) | (<.001) | (.02) | (.86) | (.56) | (.63) | (.26) | (.33) | (.10) | (.08) | (.80) | (.63) | (.69) |
| 2. Amplitude of SCRs |  | -0.04 | -0.21 | 0.15 | -0.08 | 0.00 | 0.08 | -0.14 | -0.10 | 0.02 | 0.19 | -0.14 | -0.08 | -0.04 |
|  |  | (.77) | (.12) | (.27) | (.54) | (.99) | (.56) | (.31) | (.44) | (.87) | (.16) | (.31) | (.54) | (.79) |
| 3. Rise Time of SCRs |  |  | 0.24 | 0.02 | 0.11 | 0.17 | -0.02 | 0.12 | -0.10 | 0.46 | 0.10 | -0.16 | 0.09 | 0.29 |
|  |  |  | (.07) | (.89) | (.43) | (.22) | (.86) | (.38) | (.48) | (<.001) | (.48) | (.23) | (.52) | (.03) |
| 4. Recovery Time of SCRs |  |  |  | -0.20 | -0.05 | 0.16 | 0.03 | 0.09 | -0.09 | 0.25 | 0.01 | -0.1 | 0.01 | 0.01 |
|  |  |  |  | (.11) | (.72) | (.20) | (.83) | (.50) | (.46) | (.05) | (.96) | (.45) | (.93) | (.96) |
| 5. Pupil Diameter |  |  |  |  | 0.00 | -0.15 | -0.15 | 0.01 | -0.07 | -0.00 | -0.14 | 0.13 | -0.15 | 0.12 |
|  |  |  |  |  | (.97) | (.19) | (.19) | (.91) | (.55) | (.97) | (.24) | (.27) | (.21) | (.30) |
| 6. Joystick Movement |  |  |  |  |  | 0.44 | 0.10 | -0.13 | -0.15 | 0.11 | -0.06 | 0.04 | 0.45 | 0.46 |
|  |  |  |  |  |  | (<.001) | (.40) | (.25) | (.20) | (.32) | (.60) | (.72) | (<.001) | (<.001) |
| 7. Awe-ratings |  |  |  |  |  |  | 0.15 | -0.08 | -0.22 | 0.14 | -0.05 | 0.04 | 0.51 | 0.46 |
|  |  |  |  |  |  |  | (.21) | (.48) | (.06) | (.24) | (.66) | (.72) | (<.001) | (<.001) |
| 8. Amusement-ratings |  |  |  |  |  |  |  | 0.11 | 0.01 | -0.03 | 0.06 | -0.24 | 0.12 | 0.25 |
|  |  |  |  |  |  |  |  | (.34) | (.91) | (.83) | (.62) | (.04) | (.30) | (.03) |
| 9. Small Self |  |  |  |  |  |  |  |  | 0.60 | 0.05 | 0.00 | -0.05 | -0.09 | 0.09 |
|  |  |  |  |  |  |  |  |  | (<.001) | (.64) | (.98) | (.68) | (.43) | (.42) |
| 10. Self-boundary |  |  |  |  |  |  |  |  |  | -0.07 | 0.01 | 0.04 | -0.1 | -0.07 |
|  |  |  |  |  |  |  |  |  |  | (.53) | (.94) | (.71) | (.38) | (.56) |
| 11. Perceived Uncertainty |  |  |  |  |  |  |  |  |  |  | -0.09 | 0.15 | 0.14 | 0.05 |
|  |  |  |  |  |  |  |  |  |  |  | (.43) | (.18) | (.24) | (.69) |
| 12. Life Satisfaction |  |  |  |  |  |  |  |  |  |  |  | -0.67 | -0.04 | -0.07 |
|  |  |  |  |  |  |  |  |  |  |  |  | (<.001) | (.75) | (.54) |
| 13. Subjective Stress |  |  |  |  |  |  |  |  |  |  |  |  | 0.04 | -0.04 |
|  |  |  |  |  |  |  |  |  |  |  |  |  | (.73) | (.75) |
| 14. Supernatural Agency |  |  |  |  |  |  |  |  |  |  |  |  |  | 0.56 |
|  |  |  |  |  |  |  |  |  |  |  |  |  |  | (<.001) |
| 15. Supernatural Non-agency |  |  |  |  |  |  |  |  |  |  |  |  |  |  |
| *Note*. Values in parenthesis indicate *p*-value. | | |  |  |  |  |  |  |  |  |  |  |  |  |

Table S5.

*Zero-order Correlations among Variables (Neutral Condition)*

| Variables | 2 | 3 | 4 | 5 | 6 | 7 | 8 | 9 | 10 | 11 | 12 | 13 | 14 | 15 |
| --- | --- | --- | --- | --- | --- | --- | --- | --- | --- | --- | --- | --- | --- | --- |
| 1. Number of SCRs | 0.08 | -0.21 | -0.35 | 0.03 | -0.09 | -0.03 | -0.09 | 0.02 | -0.07 | 0.06 | -0.03 | -0.07 | -0.03 | 0.01 |
|  | (.62) | (.16) | (.02) | (.79) | (.44) | (.81) | (.44) | (.86) | (.55) | (.61) | (.81) | (.52) | (.77) | (.95) |
| 2. Amplitude of SCRs |  | 0.01 | -0.46 | 0.13 | 0.12 | 0.18 | 0.15 | -0.09 | -0.21 | 0.10 | 0.15 | -0.03 | 0.27 | 0.24 |
|  |  | (.93) | (<.001) | (.38) | (.45) | (.25) | (.32) | (.55) | (.18) | (.53) | (.32) | (.82) | (.08) | (.13) |
| 3. Rise Time of SCRs |  |  | 0.41 | 0.00 | -0.11 | -0.34 | 0.22 | 0.07 | 0.09 | 0.02 | -0.00 | 0.01 | 0.04 | 0.13 |
|  |  |  | (.01) | (.99) | (.46) | (.02) | (.15) | (.63) | (.54) | (.90) | (.98) | (.97) | (.82) | (.42) |
| 4. Recovery Time of SCRs |  |  |  | -0.10 | 0.11 | -0.03 | 0.24 | -0.07 | -0.04 | 0.11 | 0.16 | -0.19 | 0.14 | -0.03 |
|  |  |  |  | (.48) | (.46) | (.84) | (.10) | (.65) | (.80) | (.46) | (.27) | (.19) | (.35) | (.86) |
| 5. Pupil Diameter |  |  |  |  | -0.07 | -0.13 | -0.04 | 0.20 | 0.13 | 0.07 | -0.13 | 0.18 | -0.06 | -0.21 |
|  |  |  |  |  | (.57) | (.27) | (.72) | (.08) | (.26) | (.55) | (.25) | (.11) | (.63) | (.07) |
| 6. Joystick Movement |  |  |  |  |  | 0.54 | 0.10 | -0.24 | -0.15 | 0.27 | 0.11 | 0 | 0.62 | 0.59 |
|  |  |  |  |  |  | (<.001) | (.40) | (.04) | (.18) | (.02) | (.36) | (.97) | (<.001) | (<.001) |
| 7. Awe-ratings |  |  |  |  |  |  | 0.15 | -0.18 | -0.19 | 0.32 | 0.06 | -0.08 | 0.31 | 0.64 |
|  |  |  |  |  |  |  | (.19) | (.12) | (.09) | (<.001) | (.61) | (.51) | (.01) | (<.001) |
| 8. Amusement-ratings |  |  |  |  |  |  |  | 0.09 | 0.18 | -0.20 | 0.03 | -0.12 | 0.16 | 0.19 |
|  |  |  |  |  |  |  |  | (.43) | (.11) | (.08) | (.76) | (.32) | (.18) | (.11) |
| 9. Small Self |  |  |  |  |  |  |  |  | 0.61 | -0.17 | 0.01 | -0.05 | -0.08 | -0.28 |
|  |  |  |  |  |  |  |  |  | (<.001) | (.14) | (.95) | (.66) | (.48) | (.02) |
| 10. Self-boundary |  |  |  |  |  |  |  |  |  | -0.35 | -0.12 | 0.03 | -0.16 | -0.23 |
|  |  |  |  |  |  |  |  |  |  | (<.001) | (.29) | (.80) | (.18) | (.05) |
| 11. Perceived Uncertainty |  |  |  |  |  |  |  |  |  |  | 0.13 | 0.11 | 0.08 | 0.23 |
|  |  |  |  |  |  |  |  |  |  |  | (.25) | (.32) | (.51) | (.05) |
| 12. Life Satisfaction |  |  |  |  |  |  |  |  |  |  |  | -0.59 | -0.05 | -0.07 |
|  |  |  |  |  |  |  |  |  |  |  |  | (<.001) | (.64) | (.55) |
| 13. Subjective Stress |  |  |  |  |  |  |  |  |  |  |  |  | 0.12 | 0.09 |
|  |  |  |  |  |  |  |  |  |  |  |  |  | (.32) | (.46) |
| 14. Supernatural Agency |  |  |  |  |  |  |  |  |  |  |  |  |  | 0.52 |
|  |  |  |  |  |  |  |  |  |  |  |  |  |  | (<.001) |
| 15. Supernatural Non-agency |  |  |  |  |  |  |  |  |  |  |  |  |  |  |
| *Note*. Values in parenthesis indicate *p*-value. | | |  |  |  |  |  |  |  |  |  |  |  |  |

Table S6.

*Results of Mean Comparisons of Self-report Variables between Conditions Using Hierarchical Bayesian Method*

| Dependent Variables | | Estimate | 95% CI | | R-hat | Bulk ESS | Tail ESS |
| --- | --- | --- | --- | --- | --- | --- | --- |
|  |  |  | Lower | Upper |  |  |  |
| Self-report Awe | |  |  |  |  |  |  |
|  | Awe_contrast | 3.18 | 2.98 | 3.37 | 1.00 | 67,870 | 32,806 |
|  | Amusement_contrast | 0.64 | 0.44 | 0.84 | 1.00 | 68,681 | 31,922 |
| Self-report Amusement | |  |  |  |  |  |  |
|  | Awe_contrast | 0.27 | –0.08 | 0.63 | 1.00 | 63,168 | 32,394 |
|  | Amusement_contrast | 1.53 | 1.18 | 1.89 | 1.00 | 66,474 | 31,593 |
| Small Self | |  |  |  |  |  |  |
|  | Awe_contrast | –1.93 | –2.17 | –1.69 | 1.00 | 67,562 | 32,365 |
|  | Amusement_contrast | –0.53 | –0.77 | –0.29 | 1.00 | 66,695 | 32,405 |
| Self-boundary | |  |  |  |  |  |  |
|  | Awe_contrast | –1.61 | –1.92 | –1.29 | 1.00 | 66,991 | 31,179 |
|  | Amusement_contrast | –0.98 | –1.29 | –0.67 | 1.00 | 69,462 | 31,357 |
| Perceived Uncertainty | |  |  |  |  |  |  |
|  | Awe_contrast | 0.53 | 0.21 | 0.85 | 1.00 | 65,190 | 32,239 |
|  | Amusement_contrast | 1.89 | 1.57 | 2.21 | 1.00 | 62,890 | 31,164 |
| Life Satisfaction | |  |  |  |  |  |  |
|  | Awe_contrast | –0.03 | –0.11 | 0.04 | 1.00 | 25,170 | 27,743 |
|  | Amusement_contrast | –0.07 | –0.14 | 0.01 | 1.00 | 25,408 | 26,763 |
| Subjective Stress | |  |  |  |  |  |  |
|  | Awe_contrast | –0.12 | –0.54 | 0.29 | 1.00 | 25,234 | 27,049 |
|  | Amusement_contrast | 0.11 | –0.30 | 0.53 | 1.00 | 25,100 | 26,106 |
| Supernatural Agency | |  |  |  |  |  |  |
|  | Awe_contrast | 1.39 | 1.13 | 1.64 | 1.00 | 67,776 | 33,210 |
|  | Amusement_contrast | 0.25 | –0.00 | 0.51 | 1.00 | 67,389 | 31,274 |
| Supernatural Non-gency | |  |  |  |  |  |  |
|  | Awe_contrast | 2.86 | 2.50 | 3.21 | 1.00 | 70,531 | 31,497 |
|  | Amusement_contrast | –0.27 | –0.62 | 0.09 | 1.00 | 67,594 | 31,879 |
| *Note*. The l- and u-95% CI indicates lower and upper bounds of 95% credible intervals, respectively; ESS: effective sample sizes. | | | | | | | |

Table S7.

*WAICs and estimated coefficients of variables for three additional models for physiological measurements that showed lower wAIC than simple models.*

| Dependent Variable | Selected Model | Lowest wAIC | Mean wAIC | Mean wAIC (simple model) | *β*_1_ | *β*_2_ | *β*_3_ | *β*_4_ | *β*_5_ | *β*_6_ |
| --- | --- | --- | --- | --- | --- | --- | --- | --- | --- | --- |
| Number of SCRs | Order | 1881.22 | 1881.62 | 1890.40 | 1.47  [1.09, 1.86] awe_contrast | 0.77  [0.38, 1.15] amusement_ contrast | -0.51  [-0.82, -0.20] order | -0.11  [-0.88, 0.64] awe_contrast*order | -0.62  [-1.38, 0.13] amusement_ contrast*order | － |
|  | Control | 1867.53 | 1867.98 |  | 1.24  [0.81, 1.66] awe_contrast | 0.43  [-0.03, 0.88] amusement_ contrast | 0.14  [0.03, 0.24] amusement | 0.15  [0.03, 0.27] fear | － | － |
| Amplitude of SCRs | Control | 1605.34 | 1605.87 | 1615.88 | 0.14  [-0.02, 0.30] awe_contrast | 0.06  [-0.11, 0.23] amusement_ contrast | 0.02  [-0.02, 0.05] amusement | 0.03  [-0.01, 0.07] fear | － | － |
| Rise Time of SCRs | Order | 90.29 | 90.60 | 96.38 | -0.07  [-0.12, -0.02] awe_contrast | -0.01  [-0.06, 0.05] amusement_ contrast | 0.05  [0.01, 0.09] order | 0.06  [-0.05, 0.16] awe_contrast*order | 0.10  [-0.01, 0.21] amusement_ contrast*order | － |
| Recovery Time of SCRs | Control | 2643.24 | 2643.52 | 2662.10 | -0.38  [-0.63, -0.12] awe_contrast | -0.04  [-0.32, 0.23] amusement_ contrast | -0.01  [-0.07, 0.04] amusement | -0.07  [-0.13, -0.01] fear | － | － |
|  | Demographic | 2655.35 | 2655.63 |  | -0.50  [-0.73, -0.27] awe_contrast | -0.16  [-0.39, 0.09] amusement_ contrast | 0.05  [-0.00, 0.11] age | -0.10  [-0.46, 0.25] gender | -0.08  [-0.13, -0.02] awe_contrast* age | -0.19  [-0.53, 0.14] awe_contrast*gender |
| Pupil Diameter | Order | -247.33 | -246.70 | -167.90 | 0.13  [0.09, 0.17] awe_contrast | 0.52  [0.49, 0.56] amusement_ contrast | -0.13  [-0.16, -0.10] order | -0.04  [-0.12, 0.03] awe_contrast*order | -0.10  [-0.17, -0.02] amusement_ contrast*order | － |
| *Note*. ﻿Mean wAIC indicates the average value of wAIC of 20 sampling runs per model, shifting the random number seed from 1 to 20; The model with the lowest wAIC value among the models with significantly lower wAIC than the simple models was used for parameter estimation; The expected a posteriori (EAP) estimates and 95% credible intervals (in square brackets) are shown for each independent variable; Awe_contrast (Amusement_contrast) was coded 2/3 for the awe (amusement) condition and –1/3 for the other conditions; Order was coded -1/2 for the first video and 1/2 for the second video; Gender was coded -1/2 for male and 1/2 for female; The values of amplitude, rise time, and recovery time of SCRs were logarithmically transformed. | | | | | | | | | | |

# References

1. Faul, F., Erdfelder, E., Lang, A.-G. & Buchner, A. G*Power 3: a flexible statistical power analysis program for the social, behavioral, and biomedical sciences. *Behav. Res. Methods* **39**, 175–191 (2007).

2. Takano, R. & Nomura, M. Awe liberates the feeling that “my body is mine.” *Cogn. Emot.* **35**, 738–744 (2021).

3. Gordon, A. M. *et al.* The dark side of the sublime: distinguishing a threat-based variant of awe. *J. Pers. Soc. Psychol.* **113**, 310–328 (2017).

4. Cohen, S., Kamarck, T. & Mermelstein, R. A global measure of perceived stress. *J. Health Soc. Behav.* **24**, 385–396 (1983).

5. Sumi, K. Reliability and validity of the Japanese version of the Perceived Stress Scale. *Japanese Journal of Health Psychology* **19**, 44–53 (2006).

6. Diener, E., Emmons, R. A., Larsen, R. J. & Griffin, S. The Satisfaction With Life Scale. *J. Pers. Assess.* **49**, 71–75 (1985).

7. Uchida, Y., Kitayama, S., Mesquita, B., Reyes, J. A. S. & Morling, B. Is perceived emotional support beneficial? Well-being and health in independent and interdependent cultures. *Pers. Soc. Psychol. Bull.* **34**, 741–754 (2008).

8. Watanabe, S. Asymptotic equivalence of Bayes cross validation and widely applicable information criterion in singular learning theory. *J. Mach. Learn. Res.* **11**, 3571–3594 (2010).

9. Bai, Y. *et al.* Awe, the diminished self, and collective engagement: universals and cultural variations in the small self. *J. Pers. Soc. Psychol.* **113**, 185–209 (2017).
